# Supplementary material for: Single-cell transcriptomics of human cholesteatoma identifies an activin A-producing osteoclastogenic fibroblast subset inducing bone destruction
Source: Nat Commun. 2023 Aug 3;14:4417. doi: 10.1038/s41467-023-40094-3 (PMC10400591; doi:10.1038/s41467-023-40094-3)
Supplement: Supplementary file 1 — Supplementary Information [file 41467_2023_40094_MOESM1_ESM.pdf]

Supplementary Information

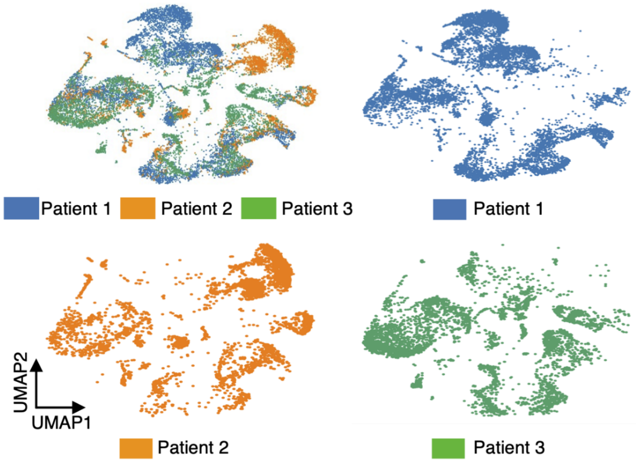

Supplementary Fig. S1. scRNA-seq images from the patients.

UMAP plot of the scRNA-seq data labeled by patient and the merged images.

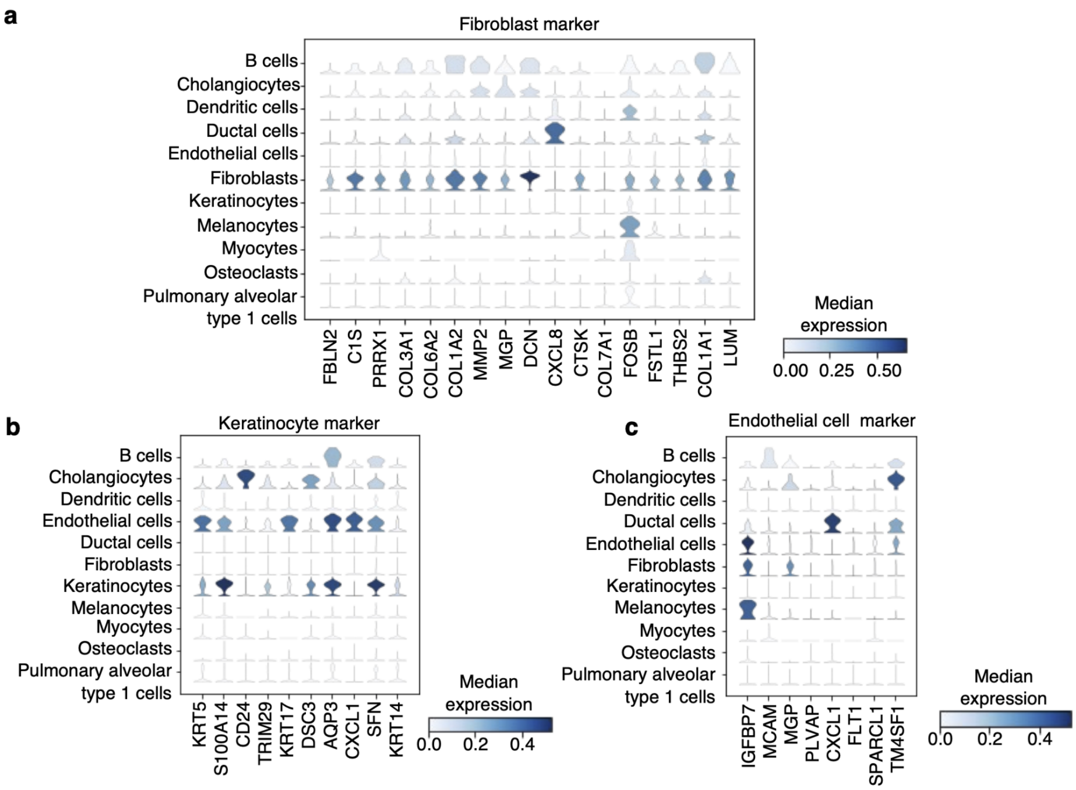

Supplementary Fig. S2. Markers in each cell type.

(a) Expression of fibroblast marker in each cluster. (b) Expression of keratinocyte marker in each cluster. (c) Expression of endothelial cell marker in each cluster.

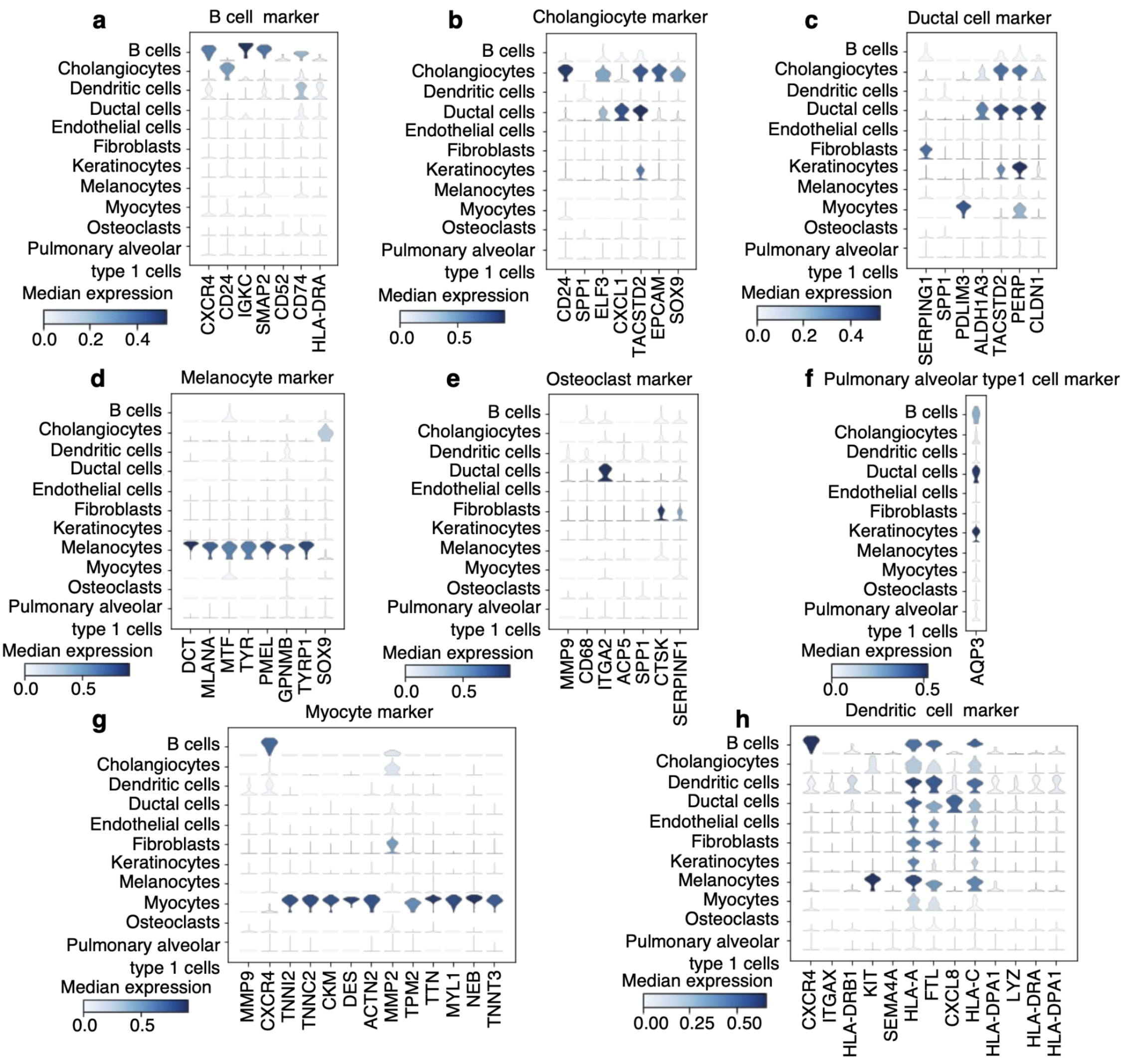

Supplementary Fig. S3. Markers of each cell type.

(a) Expression of B cell marker in each cluster. (b) Expression of cholangiocyte marker in each cluster. (c) Expression of ductal cell marker in each cluster. (d) Expression of melanocyte marker in each cluster. (e) Expression of osteoclast marker in each cluster. (f) Expression of pulmonary alveolar type 1 cell marker in each cluster. (g) Expression of myocyte marker in each cluster. (h) Expression of dendritic cell marker in each cluster.

a

Cluster	Chi square value by condition	*P*-value	Main condition
1	3153.664	0	Cholesteatoma
5	2478.13212	0	Cholesteatoma
3	1633.3608	0	Skin
4	1561.76329	0	Skin
6	1162.62985	8.07E-255	Skin
2	1010.10961	1.14E-221	Skin
0	834.318438	1.87E-183	Cholesteatoma
10	203.025616	4.57E-46	Cholesteatoma
12	104.640195	1.46E-24	Skin
9	67.2726064	2.36E-16	Cholesteatoma
13	63.7133376	1.44E-15	Cholesteatoma
14	61.4089242	4.64E-15	Skin
7	50.1389454	1.43E-12	Skin
11	44.3031324	2.81E-11	Skin
15	40.4749803	1.99E-10	Skin
8	4.57628724	0.03241738	Skin
16	1.09699597	0.29492631	Cholesteatoma

b

Cluster	Chi square value by patients	*P*-value
3	6117.07016	0
1	2162.58905	0
2	787.07178	1.23E-171
0	779.803255	4.65E-170
7	398.608508	2.77E-87
11	355.674257	5.84E-78
9	276.646563	8.45E-61
10	257.202256	1.41E-56
6	248.844214	9.21E-55
12	163.771261	2.74E-36
8	161.930313	6.88E-36
5	153.245735	5.29E-34
15	148.714326	5.09E-33
4	80.8136627	2.83E-18
14	69.3714413	8.63E-16
13	66.7906529	3.14E-15
16	40.1564255	1.91E-09

c

$$\text{C (Chi square value index)} = \frac{\text{A (Disease specificity)}}{\text{B (Sample bias)}}$$

Cluster	Chi square value index	Chi square value by condition	Chi square value by patients
4	19.325	1561.763	80.814
5	16.171	2478.132	153.246
6	4.672	1162.630	248.844
1	1.458	3153.664	2162.589
2	1.283	1010.110	787.072

Supplementary Fig. S4. Quantification of disease specificity and sample bias.

(a) The chi-square test was performed for each cluster according to sample condition using the Python package SciPy. The chi-square values of each cluster are shown in increasing order, which showed the disease specificity of each cluster. (b) The chi-square test was performed on each cluster for patients using the Python package SciPy. The chi-square values of each cluster are shown in increasing order, which showed the sample

bias of each cluster. (c) The chi-square value index was calculated to identify clusters with high disease specificity and low sample bias. The chi-square value of disease specificity was divided by the chi-square value of sample bias. This index was large in the fibroblast cluster compared to other cell type clusters.

a

Name	*P*-value	Moran's test statistic	Moran's I
CDH19	0	133.937989	0.4898721
COL10A1	0	133.184153	0.4923902
COL11A1	0	132.462384	0.4897501
COL8A1	0	110.634711	0.4090224
PI16	0	109.049125	0.4031867
GLIS3	0	105.092946	0.3884987
PCOLCE2	0	103.995327	0.3843466
LAMP5	0	102.203102	0.3775872
CILP	0	101.934503	0.3768565
EGFL6	0	97.6672823	0.3609666

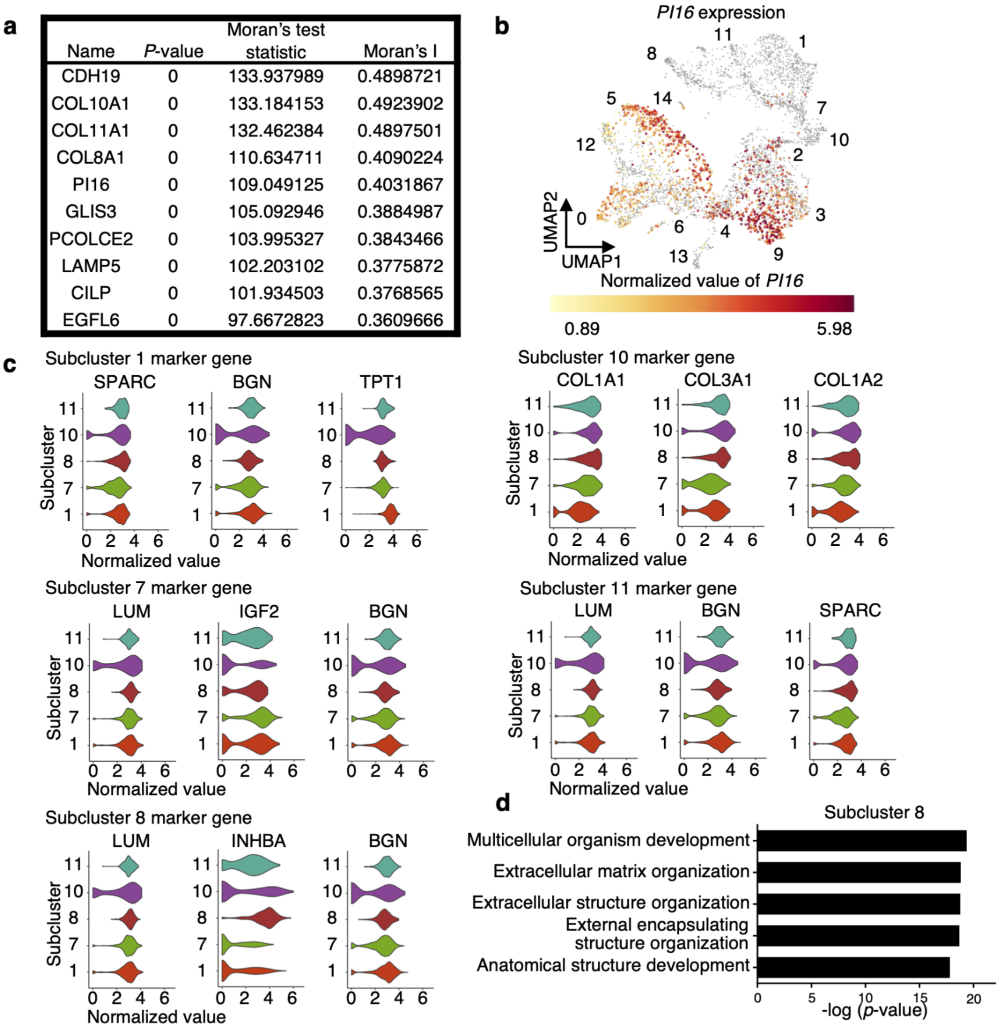

Supplementary Fig. S5. Marker genes in fibroblast subclusters.

(a) The top 10 marker genes reflecting pseudotime trajectory. (b) *PI16* expression in fibroblast subclusters. (c) Marker genes in cholesteatoma subclusters. The top three marker genes of each cluster are shown. *INHBA* was identified as a marker gene of subcluster 8 in cholesteatoma fibroblasts. (d) GO biological pathway upregulated in subcluster 8. Statistical significance was determined using the Moran's I test (a). The

enrichment analysis was conducted through gprofiler[59] integrated with scanpy v1.9, as well as DAVID[60] (accessed on 7/8 2021) in (d).

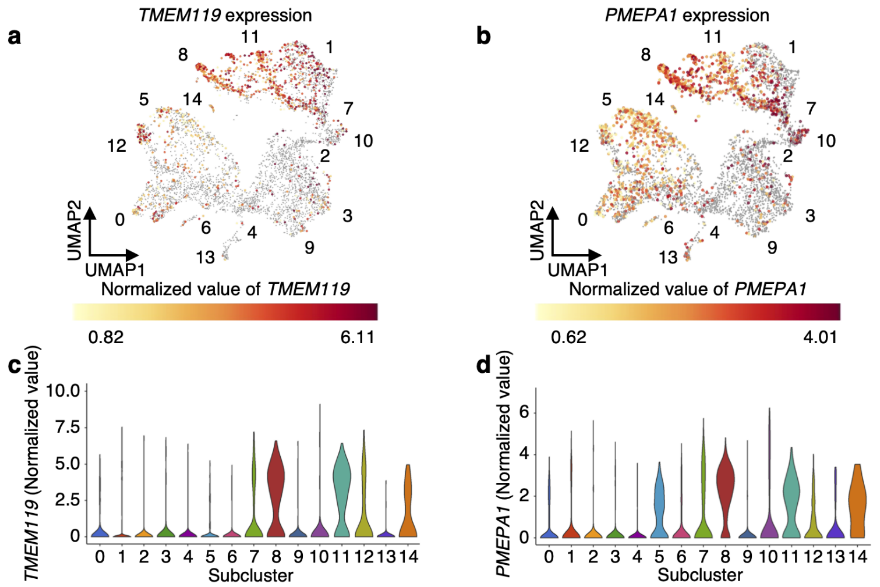

Supplementary Fig. S6. Surface marker candidate genes in cholesteatoma-specific fibroblasts

(a) *TMEM119* expression in fibroblast subclusters. (b) *PMEPA1* expression in fibroblast subclusters. (c) Violin plots of *TMEM119* expression in each cluster. (d) Violin plots of *PMEPA1* expression in each cluster.

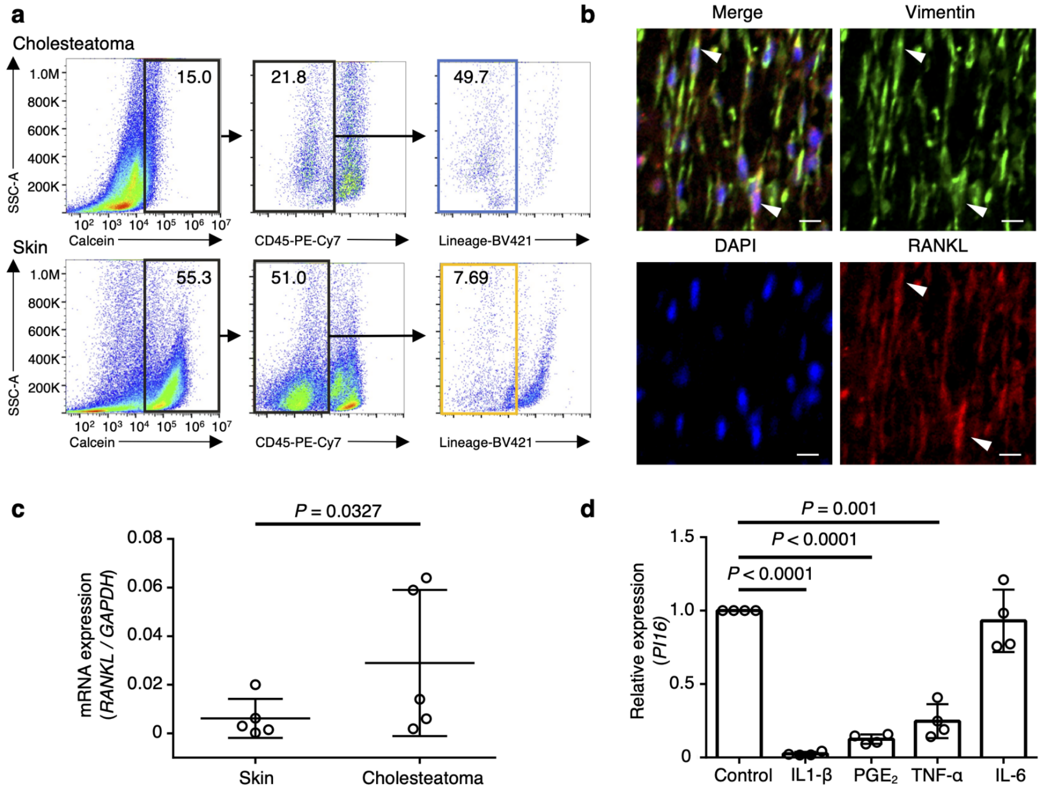

Supplementary Fig. S7. Analysis of human fibroblasts.

(a) Gating strategy for human fibroblasts. Cholesteatoma and control skin fibroblasts were sorted as calcein^{+} CD45^{-} E-cadherin^{-} CD31^{-} cells. (b) Colocalization of vimentin and RANKL immunofluorescence indicating that fibroblasts in the perimatrix expressed RANKL. RANKL was labeled with Alexa Fluor 568 and vimentin was labeled with Alexa Fluor 488. Scale bars: 10 μm. (c) RANKL mRNA expression level, normalized relative to GAPDH mRNA expression, was significantly higher in cholesteatoma than control skin fibroblasts (n = 5). (d) IL-1β, PGE_2, and TNF-α inhibited *PI16* expression in human primary skin fibroblasts (n = 4). The exact p value between the control and IL-1β

group was 0.00004; that between the control and the PGE_2 group was 0.00008. Data represent means ± SDs. Statistical significance was determined using the ratio paired two-tailed t-test (c) and two-tailed unpaired t-test (d). Source data are provided as a Source Data file.

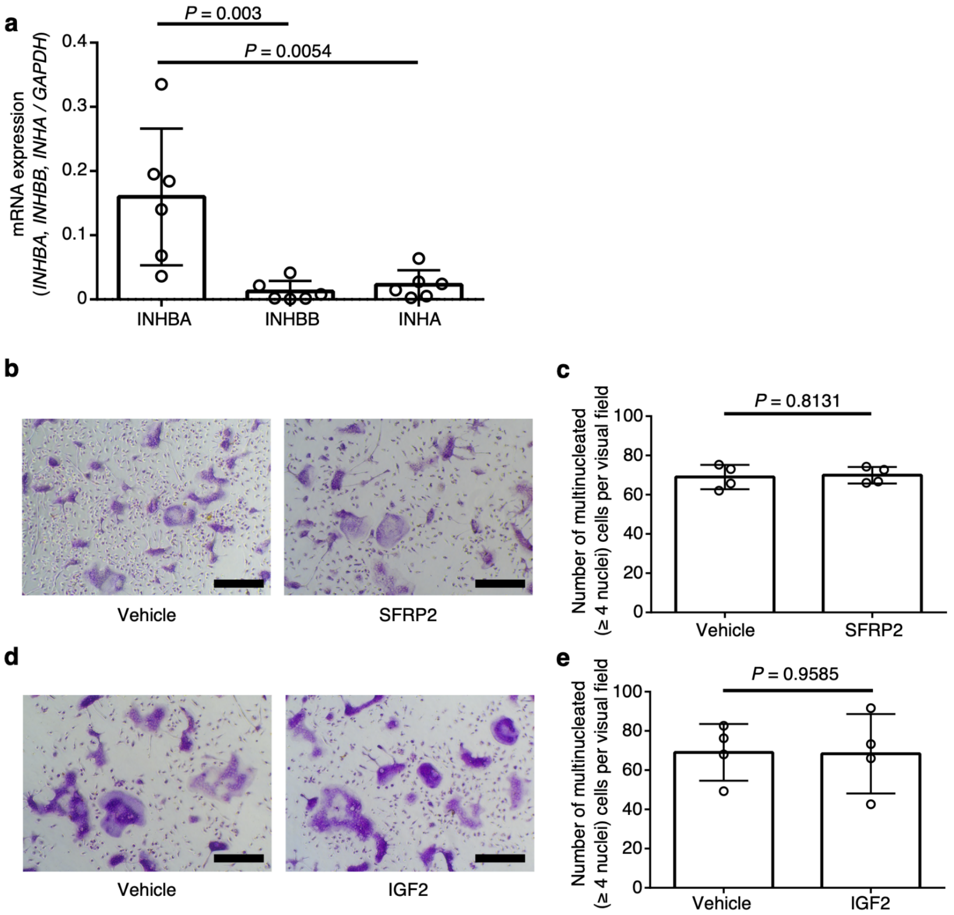

Supplementary Fig. S8. Expression of INHBA, INHBB, and INHA mRNA, and the effect of SFRP2 and IGF2 on osteoclast differentiation.

(a) *INHBA*, *INHBB*, and *INHA* mRNA expression levels are shown. *INHBA* normalized relative to *GAPDH* expression was significantly upregulated compared to *INHBB* and *INHA* in cholesteatoma fibroblasts (n = 6). (b) Representative images of TRAP staining in bone marrow macrophages cultured with 10 ng mL^{-1} M-CSF and 50 ng mL^{-1} RANKL. SFRP2 at 100 ng mL^{-1} did not promote osteoclast differentiation. Scale bars: 200 μm (c)

Quantification of TRAP-positive multinuclear cells within the visual field in b (n = 4 per group). (d) Representative images of TRAP staining in bone marrow macrophages cultured with 10 ng mL^{-1} M-CSF and 50 ng mL^{-1} RANKL. IGF2 at 10 ng mL^{-1} did not promote osteoclast differentiation. Scale bars: 200 μm. (e) Quantification of TRAP-positive multinuclear cells within the visual field in d (n = 4 per group). Data represent means ± SDs. Statistical significance was determined by one-way ANOVA with Tukey's post hoc multiple comparison test (a) and two-tailed unpaired t-test (c, e). Source data are provided as a Source Data file.

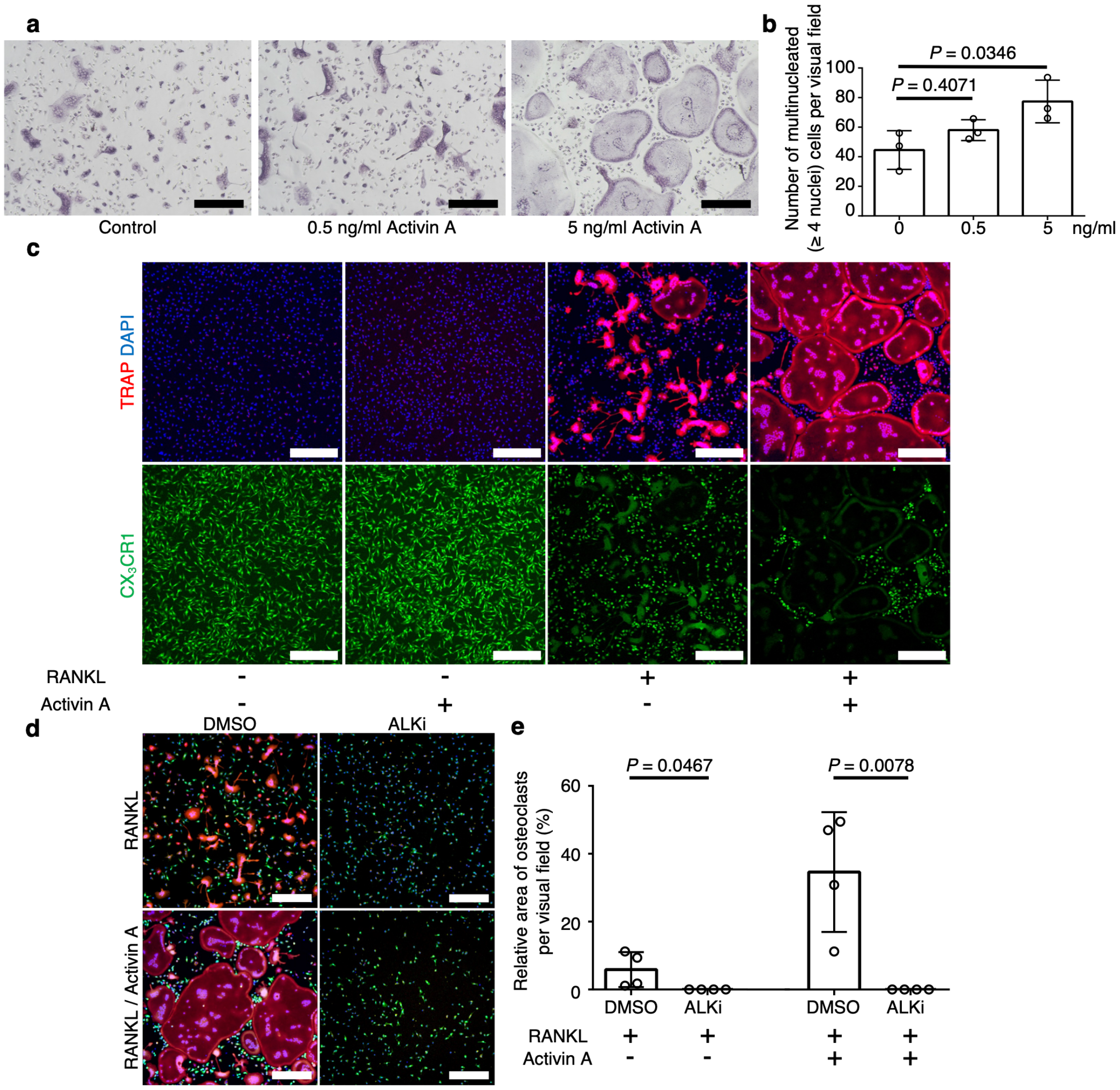

Supplementary Fig. S9. Activin A functional analysis in vitro.

(a) Representative images of TRAP staining in bone marrow macrophages cultured with 10 ng mL^{-1} M-CSF and 50 ng mL^{-1} RANKL. Activin A promoted osteoclastogenesis in a concentration-dependent manner. Scale bars: 200 μm. (b) Quantification of TRAP-positive multinuclear cells within the visual field in a (n = 3 per group). (c) Individual channel images of Fig. 4a. Bone marrow macrophages derived from CX_3CR1-

EGFP/TRAP-tdTomato mice were cultured with 10 ng mL^{-1} M-CSF. RANKL (50 ng mL^{-1}) and activin A (5 ng mL^{-1}) were added either alone or in combination. Green: CX_3CR1-$EGFP^+$ cells; red: TRAP-$tdTomato^+$ cells; blue: nuclei. Scale bars: 300 μm. (d) Representative images of TRAP-positive cells derived from double transgenic mouse bone marrow macrophages cultured with 10 ng mL^{-1} M-CSF and RANKL (50 ng mL^{-1}). Activin A (5 ng mL^{-1}) and ALK-4 inhibitor (10 μM) were added either alone or in combination. Green: CX_3CR1-$EGFP^+$ cells; red: TRAP-$tdTomato^+$ cells; blue: nuclei. Scale bars: 300 μm. (e) Quantification of TRAP-positive area within the visual field. (n = 4 per group). Data represent means ± SDs. Statistical significance was determined by one-way ANOVA with Tukey's post hoc multiple comparison test (b) and two-tailed unpaired t-test (e). Source data are provided as a Source Data file.

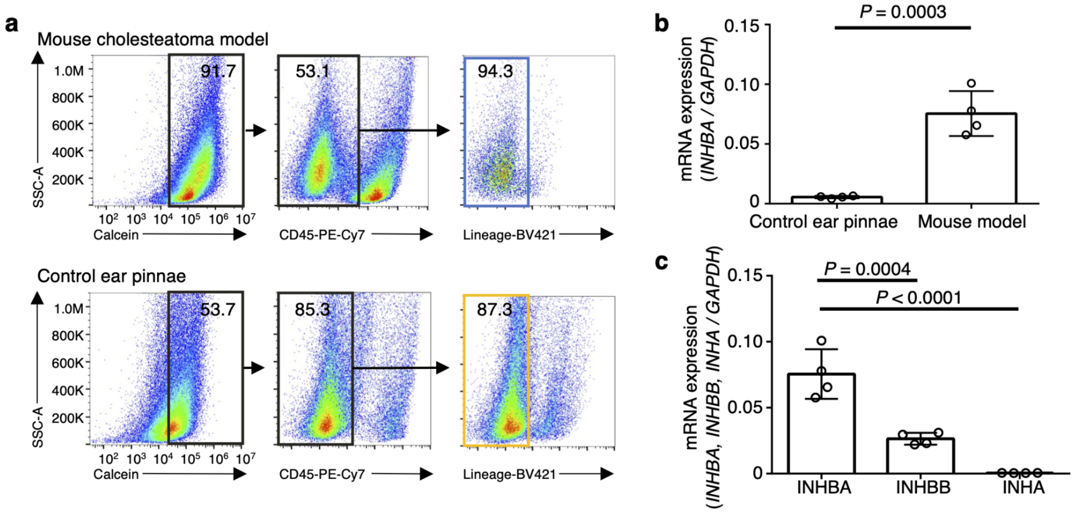

Supplementary Fig. S10. Analysis of mouse fibroblasts.

(a) Gating strategy for mouse fibroblasts. Cholesteatoma and control ear pinnae-derived mouse fibroblasts were sorted as calcein^{+} CD45^{-} E-cadherin^{-} CD31^{-} cells. (b) *INHBA* mRNA expression levels are shown. *INHBA* normalized relative to *GAPDH* mRNA expression was significantly higher in mouse cholesteatoma fibroblasts than in control mouse ear pinnae fibroblasts (n = 4). (c) *INHBA*, *INHBB*, and *INHA* mRNA expression levels are shown. *INHBA* normalized relative to *GAPDH* mRNA level was significantly upregulated compared to *INHBB* and *INHA* in cholesteatoma fibroblasts. Data represent means ± SDs. Statistical significance was determined using the two-tailed unpaired t-test (b) and one-way ANOVA with Tukey's post hoc multiple comparison test (c). The exact p value between the INHBA and INHA group was 0.00001. Source data are provided as a Source Data file.

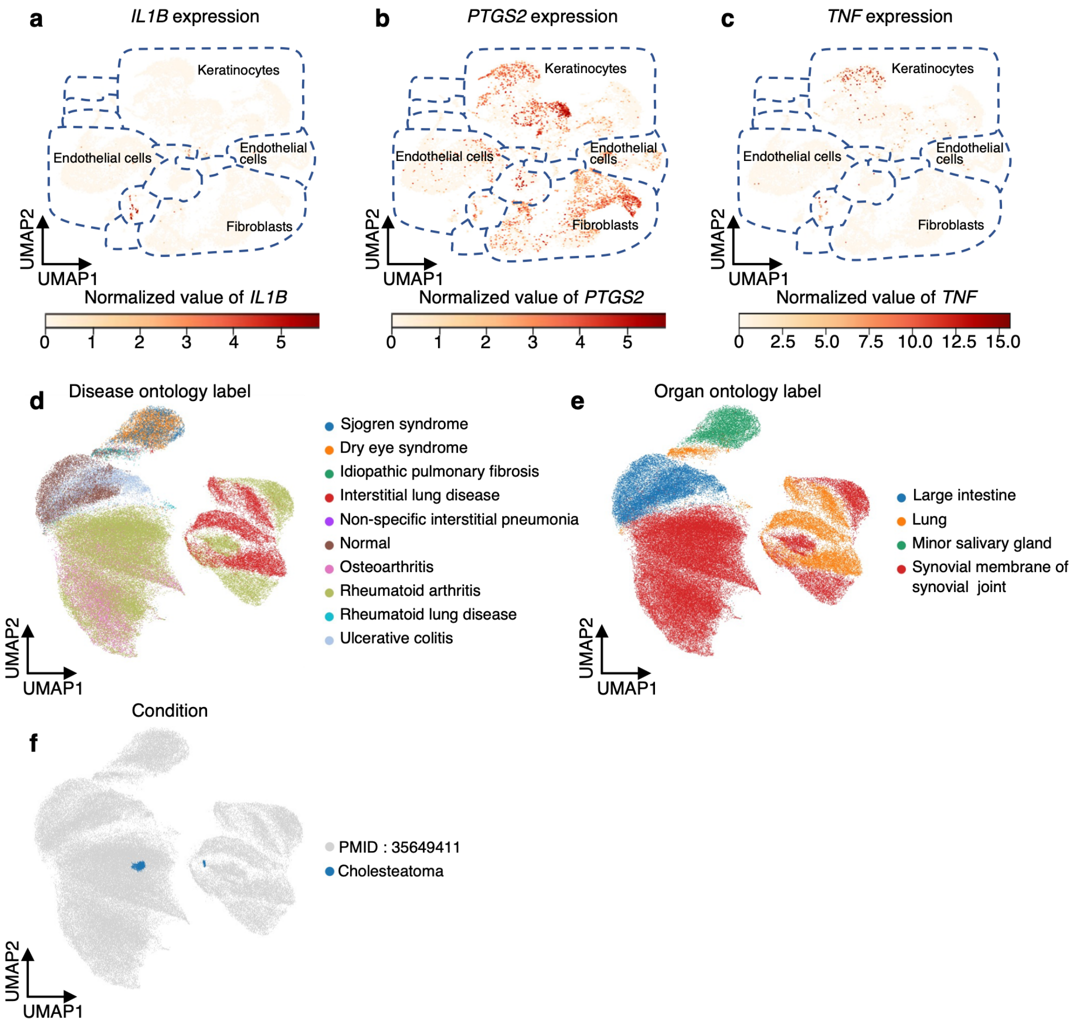

Supplementary Fig. S11. *IL1B*, *PTGS2*, and *TNF* expression in scRNA-seq and integration with public data sets.

(a) *IL1B* expression according to scRNA-seq. Cholesteatoma did not show upregulation of *IL1B* compared to skin fibroblasts. (b) *PTGS2* expression according to scRNA-seq. Cholesteatoma did not show upregulation of *PTGS2* compared to skin fibroblasts. (c) *TNF* expression according to scRNA-seq. Cholesteatoma did not show upregulation of

TNF compared to skin fibroblasts. (d–f) The integrated data analysis is displayed with disease ontology labels (d). Similarly, the integrated data are represented with organ ontology labels (e). The fibroblasts examined in our study exhibited molecular properties similar to those of the fibroblasts derived from the synovial membranes of joints affected by rheumatoid arthritis (f).

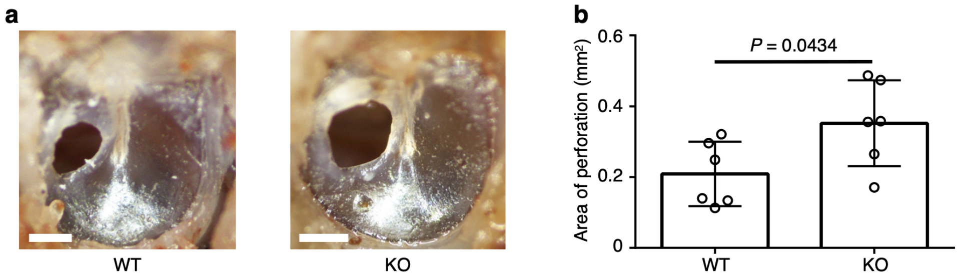

Supplementary Fig. S12. Physiological function of activin A in the tympanic membrane.

(a) After inhibition of *INHBA* expression, the tympanic membrane showed larger perforations, compared to the controls. Scale bars: 500 µm. (b) The *INHBA*-KO group showed reduced tympanic membrane wound healing, compared to the control group (n = 4). Data represent means ± SDs. Statistical significance was determined using the two-tailed unpaired t-test. Symbols represent individual mice. Source data are provided as a Source Data file.

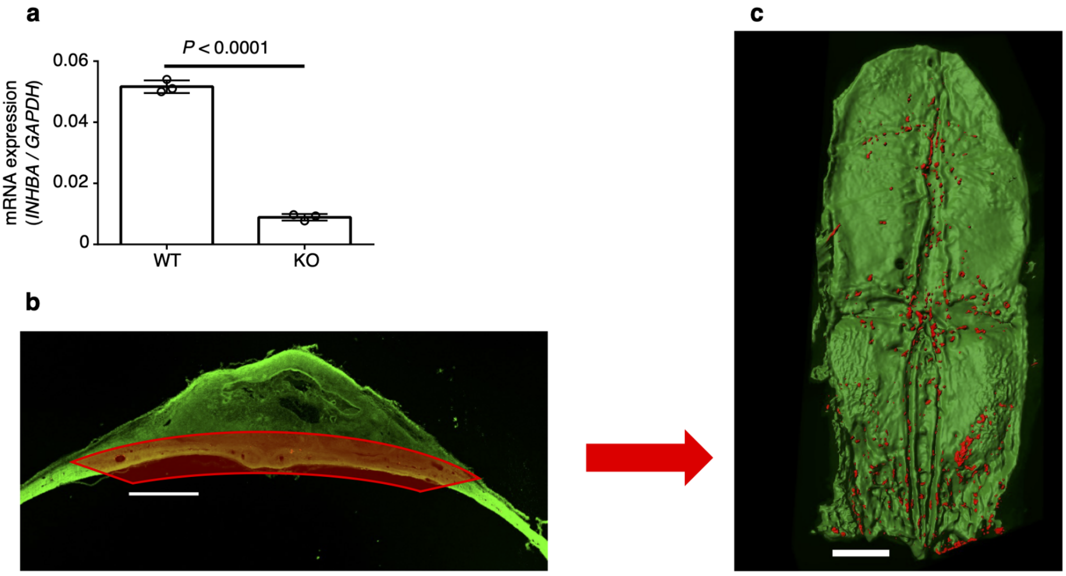

Supplementary Fig. S13. Mouse model of cholesteatoma to detect osteoclasts induced on the parietal bone surface.

(a) Reverse transcription PCR was performed in fibroblasts cultured with 800 nM 4-hydroxytamoxifen (4OHT). Fibroblasts derived from *INHBA*$^{fl/fl}$ × *Rosa26*^{CreERT2} mice showed inhibition of *INHBA* expression compared to fibroblasts derived from *INHBA*$^{fl/fl}$ × WT mice (n = 3). The exact p value was 0.000006. Data represent means ± SDs. Statistical significance was determined using the two-tailed unpaired t-test. (b) Histological sections from cholesteatoma model mice. TRAP-positive cells were labeled with tdTomato, and sections were stained with Alexa Fluor 488 NHS ester. TRAP-positive osteoclasts were induced on the parietal bone surface. Scale bars: 1000 µm. (c) Representative image of the parietal bone surface beneath the cholesteatoma mass.

TRAP-positive osteoclasts were induced on the parietal bone surface. Red, TRAP-tdTomato$^+$ cells; green, parietal bone surface. Scale bars: 1,000 μm. Symbols represent individual mice. Source data are provided as a Source Data file.
