# Supplementary material for: Single-cell transcriptomics of human cholesteatoma identifies an activin A-producing osteoclastogenic fibroblast subset inducing bone destruction
Source: Nat Commun. 2023 Aug 3;14:4417. doi: 10.1038/s41467-023-40094-3 (PMC10400591; doi:10.1038/s41467-023-40094-3)
Supplement: Supplementary file 3 — Reporting Summary [file 41467_2023_40094_MOESM3_ESM.pdf]

Corresponding author(s): Junichi Kikuta and Masaru Ishii

Last updated by author(s): Jun 8, 2023

Reporting Summary

Nature Portfolio wishes to improve the reproducibility of the work that we publish. This form provides structure for consistency and transparency in reporting. For further information on Nature Portfolio policies, see our Editorial Policies and the Editorial Policy Checklist.

Statistics

For all statistical analyses, confirm that the following items are present in the figure legend, table legend, main text, or Methods section.

n/a	Confirmed	
☐	☒	The exact sample size (n) for each experimental group/condition, given as a discrete number and unit of measurement
☐	☒	A statement on whether measurements were taken from distinct samples or whether the same sample was measured repeatedly
☐	☒	The statistical test(s) used AND whether they are one- or two-sided<br>*Only common tests should be described solely by name; describe more complex techniques in the Methods section.*
☒	☐	A description of all covariates tested
☐	☒	A description of any assumptions or corrections, such as tests of normality and adjustment for multiple comparisons
☐	☒	A full description of the statistical parameters including central tendency (e.g. means) or other basic estimates (e.g. regression coefficient) AND variation (e.g. standard deviation) or associated estimates of uncertainty (e.g. confidence intervals)
☐	☒	For null hypothesis testing, the test statistic (e.g. F, t, r) with confidence intervals, effect sizes, degrees of freedom and P value noted<br>*Give P values as exact values whenever suitable.*
☒	☐	For Bayesian analysis, information on the choice of priors and Markov chain Monte Carlo settings
☒	☐	For hierarchical and complex designs, identification of the appropriate level for tests and full reporting of outcomes
☒	☐	Estimates of effect sizes (e.g. Cohen's d, Pearson's r), indicating how they were calculated

Our web collection on statistics for biologists contains articles on many of the points above.

Software and code

Policy information about availability of computer code

Data collection	SH 800 cell sorter, BD Rhapsody Single-Cell Analysis System, Illumina NovaSeq 6000 platform, QX200 ddPCR system, NIS Elements v.5.40.00, TP800 Dice Real-Time Thermal Cycler System
Data analysis	FlowJo v.10, Quanta Software version 1.7.4, ImageJ software v.2.3.0, GraphPad Prism v.6, Imaris v.9.3.1, Scanpy v.1.9.0, Scanorama v.1.4, , Monocle 3, BioTuring BBrowser v.3.2.8. PanglaoDB [access date: 2021/08/07], All source code has been made publicly via Zenodo [https://doi.org/10.5281/zenodo.7983288].

For manuscripts utilizing custom algorithms or software that are central to the research but not yet described in published literature, software must be made available to editors and reviewers. We strongly encourage code deposition in a community repository (e.g. GitHub). See the Nature Portfolio guidelines for submitting code & software for further information.

Data

Policy information about availability of data

All manuscripts must include a data availability statement. This statement should provide the following information, where applicable:

- Accession codes, unique identifiers, or web links for publicly available datasets
- A description of any restrictions on data availability
- For clinical datasets or third party data, please ensure that the statement adheres to our policy

Single-cell RNA-sequencing data have been deposited in the NCBI Gene Expression Omnibus (GEO) database under accession number: GSE210261 [https://www.ncbi.nlm.nih.gov/geo/query/acc.cgi?acc=GSE210261]. The marker genes data for cell type annnotation are available at PanglaoDB [https://panglaodb.se/

index.html]. All other data that support the findings of this study are available within the article and its supplementary information files. Source data are provided with this paper.

Human research participants

Policy information about studies involving human research participants and Sex and Gender in Research.

Reporting on sex and gender	Sex and gender were not considered in study design because the sample size was not enough to consider sex difference.
Population characteristics	The study involves cholesteatoma sample and retroauricular skin at the incision cite from cholesteatoma patients. Sex is not considered in this study.
Recruitment	All fifteen cholesteatoma and skin specimens were collected from patients who underwent tympanomastoidectomy. Patients who had sufficient sample volume available for analysis in the experiment were recruited. The bias by excluding mild patients may had the impact of making the results of this study more serious compared to the average of whole cholesteatoma patients.
Ethics oversight	Research involving human subjects was approved by the Institutional Review Board at Osaka University with appropriate informed consent.

Note that full information on the approval of the study protocol must also be provided in the manuscript.

Field-specific reporting

Please select the one below that is the best fit for your research. If you are not sure, read the appropriate sections before making your selection.

- [x] Life sciences
- [ ] Behavioural & social sciences
- [ ] Ecological, evolutionary & environmental sciences

For a reference copy of the document with all sections, see nature.com/documents/nr-reporting-summary-flat.pdf

Life sciences study design

All studies must disclose on these points even when the disclosure is negative.

Sample size	We estimated the required sample sizes by considering variations and means, and sought to reach reliable conclusions using sample sizes that were as small as possible. Previously published results, complexity, the cost of experiments and past experience were used to determine the sample size.
Data exclusions	No data were excluded.
Replication	Experiments included sufficient sample size to ensure the reproducibility of the findings. All experiments reported in this study were independently repeated at least three times, while all attempts at replication were successful.
Randomization	For in vivo studies, mice were randomly assigned to each treatment group within each genotype. For in vitro studies, conditions were randomly assigned to each experimental condition.
Blinding	All assays were not relevant to blinding because the same author is involved in all experiments and analysis.

Reporting for specific materials, systems and methods

We require information from authors about some types of materials, experimental systems and methods used in many studies. Here, indicate whether each material, system or method listed is relevant to your study. If you are not sure if a list item applies to your research, read the appropriate section before selecting a response.

Materials & experimental systems

n/a	Involved in the study	
[ ]	[x]	Antibodies
[x]	[ ]	Eukaryotic cell lines
[x]	[ ]	Palaeontology and archaeology
[ ]	[x]	Animals and other organisms
[x]	[ ]	Clinical data
[x]	[ ]	Dual use research of concern

Methods

n/a	Involved in the study	
[x]	[ ]	ChIP-seq
[ ]	[x]	Flow cytometry
[x]	[ ]	MRI-based neuroimaging

Antibodies

Antibodies used	anti-human CD45-BV421 (304031, HI30; BioLegend, San Diego, CA, USA). 1:50 dilution, anti-human CD45-PEcy7 (304015, HI30; BioLegend). 1:50, anti-human CD31-BV421 (303123, WM59; BioLegend).1:50, and anti-mouse/human CD324-BV421 (147319, DECMA-1; BioLegend). 1:50, anti-mouse-CD45-PEcy7 (103113, 30-F11; BioLegend).1:50, anti-mouse-CD31-BV421 (102423, 390; BioLegend), 7AAD (559925, 1:50; BD Biosciences, Franklin Lakes, NJ, USA) for flow cytometry and cell sorting. anti-human activin A antibody (ab56057, Abcam, Cambridge, UK). 1:200, anti-human RANKL antibody (ab216484, Abcam). 1:100, anti-human vimentin antibody (MAB2105-SP,280618, R&D Systems, Minneapolis, MN, USA). 1:100, or anti-human CD45 antibody (FAB3791R-025, R&D Systems). 1:20, Alexa Fluor 488-conjugated goat anti-rat IgG (A11006, Invitrogen, Carlsbad, CA, USA). 1:500, Alexa Fluor 568-conjugated goat anti-rabbit IgG (A11011, Invitrogen). 1:500 for double immunofluorescene staining
Validation	All the antibodies used is commercially available and their validation statements are available on the manufacturer's website:<br><br>anti-human CD45-BV421 (304031, HI30; BioLegend, San Diego, CA, USA)<br>https://www.biolegend.com/ja-jp/products/brilliant-violet-421-anti-human-cd45-antibody-7332<br><br>anti-human CD45-PEcy7 (304015, HI30; BioLegend)<br>https://www.biolegend.com/ja-jp/products/pe-cyanine7-anti-human-cd45-antibody-1915<br><br>anti-human CD31-BV421 (303123, WM59; BioLegend)<br>https://www.biolegend.com/ja-jp/products/brilliant-violet-421-anti-human-cd31-antibody-8588<br><br>anti-mouse/human CD324-BV421 (147319, DECMA-1; BioLegend)<br>https://www.biolegend.com/ja-jp/products/brilliant-violet-421-anti-mouse-human-cd324-e-cadherin-antibody-16415<br><br>anti-mouse CD45-PEcy7 (103113, 30-F11; BioLegend)<br>https://www.biolegend.com/ja-jp/products/pe-cyanine7-anti-mouse-cd45-antibody-1903?GroupID=BLG1932<br><br>anti-mouse CD31-BV421 (102423, 390; BioLegend)<br>https://www.biolegend.com/ja-jp/products/brilliant-violet-421-anti-mouse-cd31-antibody-8599<br><br>anti-human activin A antibody (ab56057, Abcam, Cambridge, UK)<br>https://www.abcam.com/inhibin-beta-a-antibody-ab56057.html<br><br>anti-human RANKL antibody (ab216484, Abcam)<br>https://www.abcam.co.jp/rankl-antibody-ab216484.html<br><br>anti-human vimentin antibody (MAB2105-SP, R&D Systems, Minneapolis, MN, USA)<br>https://www.rndsystems.com/products/human-mouse-rat-vimentin-antibody-280618_mab2105<br><br>Alexa Fluor 488-conjugated goat anti-rat IgG (A11006, Invitrogen, Carlsbad, CA, USA)<br>https://www.thermofisher.com/antibody/product/Goat-anti-Rat-IgG-H-L-Cross-Adsorbed-Secondary-Antibody-Polyclonal/A-11006<br><br>Alexa Fluor 568-conjugated goat anti-rabbit IgG<br>https://www.thermofisher.com/antibody/product/Goat-anti-Rabbit-IgG-H-L-Cross-Adsorbed-Secondary-Antibody-Polyclonal/A-11011

Animals and other research organisms

Policy information about studies involving animals; ARRIVE guidelines recommended for reporting animal research, and Sex and Gender in Research

Laboratory animals	All mice used in this study (TRAP-tdTomato, CX3CR1-EGFP, INHBA-floxed, ROSA26-CreERT2) were derived on C57BL/6 (B6) background. INHBA-floxed mice were provided by N, Emoto. INHBA-floxed mice were bred with ROSA26-CreERT2 by us. For most of the studies, 8-12-week-old female mice were used.
Wild animals	No wild animals were used in this study.
Reporting on sex	Sex and gender were not considered in study design.
Field-collected samples	No field collected samples were used in this study.
Ethics oversight	Animal studies were approved by the Institutional Review Board of Osaka University.

Note that full information on the approval of the study protocol must also be provided in the manuscript.

Flow Cytometry

Plots

Confirm that:

- [x] The axis labels state the marker and fluorochrome used (e.g. CD4-FITC).
- [x] The axis scales are clearly visible. Include numbers along axes only for bottom left plot of group (a 'group' is an analysis of identical markers).
- [x] All plots are contour plots with outliers or pseudocolor plots.
- [x] A numerical value for number of cells or percentage (with statistics) is provided.

Methodology

Sample preparation	Cholesteatoma specimens and corresponding control skin specimens were cut into plugs, 4 mm in diameter, and treated using a Human Whole Skin Dissociation kit (Miltenyi Biotec, Bergisch Gladbach, Germany) according to the manufacturer's protocol and digested mechanically using a gentleMACS Dissociator (Miltenyi Biotec) with the program h_skin_01. Collected cell suspensions were filtered through 40-m cell strainers, centrifuged at 300 × g for 10 min at 4°C, and treated with a debris removal solution kit (Miltenyi Biotec) according to the manufacturer's protocol. The obtained cells were treated with red blood cell lysis buffer (Sigma-Aldrich, St. Louis, MO, USA) and used for scRNA-seq, flow cytometry, and cell sorting. The mass of cholesteatoma model and mouse ear pinna specimens were cut into 4mm diameter plugs, and treated with dispase (4 mg/mL) and collagenase (3 mg/mL) at 37°C for 2 h, and the obtained cells were used for flow cytometry and cell sorting.
Instrument	Cell sorter SH800 (SONY)
Software	FlowJo v.10
Cell population abundance	The abundance of positive cell population was generally above 5%.
Gating strategy	The stained samples were matched with non-stained negative controls to determine the positive and negative population. Cells were gated for singlet according to FSC-H/FSC-A. The gated cells were then gated for each desired positive or negative cells according to each determination.

- [x] Tick this box to confirm that a figure exemplifying the gating strategy is provided in the Supplementary Information.
